# Experimental models of murine atherosclerosis: does perception match reality?

**DOI:** 10.1093/cvr/cvy140

**Published:** 2018-06-05

**Authors:** Jessica M Johnston, Sheila E Francis, Endre Kiss-Toth

**Affiliations:** Department of Infection, Immunity and Cardiovascular Disease, University of Sheffield, Beech Hill Road, Sheffield S10 2RX, UK

## 1. Introduction

Murine models of atherosclerosis have been invaluable to gain mechanistic understanding of this chronic disease. Induction of atherosclerosis with relative ease in apolipoprotein E knockout (*ApoE*^*−*^^*/*^^*−*^) and low density lipoprotein receptor knockout (*Ldlr*^*−*^^*/*^^*−*^) mice fed a western-type diet to cause hyperlipidaemia has provided researchers with popular tools to manipulate and characterise the action of genes that affect atherogenesis. Seminal studies and reviews have discussed key phenotypic differences between different models and ‘target levels of hyperlipidaemia’ have also been published.^1^^,^^2^ However, despite widespread use of these models and firm beliefs about differences in severity between them, there has been no meta-analysis and critical appraisal of published literature reporting phenotypes of murine atherosclerosis, focusing on the degree of hyperlipidaemia, and aortic atheroma burden.

## 2. Methods and results

A systematic search in PubMed and Web of Knowledge with the key words: ‘atherosclerosis’, ‘western diet’, and ‘mice’ identified 483 studies between 2006 and 2018. These reports used *ApoE*^*−*^^*/*^^*−*^, *Ldlr*^*−*^^*/*^^*−*^, or proprotein convertase subtilisin/kexin type 9 overexpressing- adeno-associated virus (PCSK9-AAV8) models of experimental atherosclerosis (including bone marrow transplants, BMT), induced by western-diet (WD) feeding. We chose to assess standard WD (0.2% cholesterol) as it is the most widely used diet in atherosclerosis studies.^1^ These publications were filtered based on the content of their abstracts; those that described experiments of experimental atherosclerosis (204 papers) were considered for further assessment. Of these, articles that reported at least two of the following measures were included in the main analysis to allow for correlative comparisons; lesion area in the aortic root (numerical values and not %), % *en face* Oil-Red O staining in the whole aorta, total plasma cholesterol, and triglyceride (mg/dL) levels. These criteria yielded 144 research articles (22 used BMT of either model, 75 used *ApoE*^*−*^^*/*^^*−*^, 27 used *Ldlr*^*−*^^*/*^^*−*^, 10 used both BMT and single *ApoE*^*−*^^*/*^^*−*^ or *Ldlr*^*−*^^*/*^^*−*^, 2 used PCSK9, and the remaining 8 studies used mixed models BMT) where the mice were fed a diet for 4–28 weeks.

**Figure 1:**
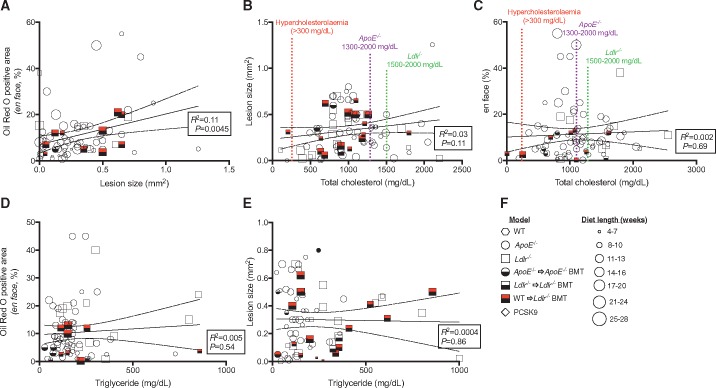
Correlation between *en face* Oil-Red O staining and aortic root lesion size vs. plasma cholesterol and triglyceride. (*A*) Correlation of *en face* Oil-Red O staining vs. lesion size. Symbols used denote different murine atherosclerosis models (refer to key in *F*). (*B*) Correlation of lesion size vs. total plasma cholesterol. Dotted lines describe classification of total plasma cholesterol as per, normal levels (<100 mg/dL),^1^^,^^3^ hypercholesterolaemia (>300 mg/dL, red)^1^, expected levels in *ApoE^−^*^/^^*−*^ and *Ldlr^−^*^/^^*−*^ mice fed western diet (1300–2000 mg/dL, purple and 1500–2000 mg/dL, green), respectively.^1^^,^^2^ (*C*) Correlation of *en face* Oil-Red O staining vs. total plasma cholesterol. Dotted lines refer to plasma cholesterol levels as in (*B*). (*D*) Correlation of *en face* Oil-Red O staining vs. plasma triglyceride. (*E*) Correlation of lesion size vs. plasma triglyceride. Linear regression and Pearson correlation coefficients were calculated (95% CI, dotted lines). *R*^2^ is reported along with level of significance. Data points represent the mean given in each publication. (*F*) Key for symbols of the different experimental models analysed.

We found that atherosclerotic burden (*en face*) vs. lesion size in the aortic root correlated strongly (*R*^2^ = 0.11, *P* = 0.0045) across the studies (*Figure 1A*). We then selected publications (*N* = 72) that used the most common duration of atherogenic diet (8–16 weeks) and tested for correlation between atherosclerotic burden in the aorta (*en face*) or lesion size at the aortic sinus vs. total plasma cholesterol levels. This analysis showed a weak trend towards positive correlation between lesion size and cholesterol (*R*^2^ = 0.03, *P* = 0.11; *Figure 1B*), with no correlation between the degree of *en face* positive staining and cholesterol (*Figure 1C*). Similarly, plasma triglyceride levels did not correlate with atherosclerotic burden (*Figure 1D*) or lesion size either (*Figure 1E*).

Further, we found no increase in atherosclerotic burden over time in any of the models. The lack of segregation for the different models and substantial variability between studies using the same model and same length of diet was also notable. Surprisingly, approximately 70% of studies fell outside of the 95% confidence interval even for the most highly associated parameters (*en face* vs. lesion size). Finally, plasma cholesterol levels in the vast majority of the studies were hypercholesterolaemic (>300 mg/dL)^1^ but more than 80% were below levels commonly referred to as ‘expected’ in *ApoE*^*−*^^*/*^^*−*^ (>1300 mg/dL) and *Ldlr*^*−*^^*/*^^*−*^ (>1500 mg/dL) mice fed a western-type diet.^1^^,^^2^ Whilst a previous study reported opposite effects on atherosclerotic burden in the aorta vs. aortic root between *Ldlr*^*−*^^*/*^^*−*^ and *Ldlr*^*−*^^*/*^^*−*^ BMT mice,^4^ we did not find evidence for such skewing in the data analysed (*Figure 1A*).

## 3. Conclusions

Contrary to perception in the field, the reality is that the degree of hyperlipidaemia and measured burden of atherosclerosis is highly variable between experimental studies and in this analysis, there is no evidence for a time-dependent increase in atherogenesis within or overall across the various models, despite the number of reports showing increase in atheroma burden between two time points in the same study.^5–8^ We also found no discrimination between different models in the degree of atherosclerosis in the published literature and speculate that this is unlikely to be solely due to technical differences in analysis and that environmental differences between animal facilities including housing strategies, in-house pathogens and differences in microbiomes are likely to be critical.^9–11^ Thus, we believe that well controlled and sufficiently powered experiments should be interpreted on their own merits, rather than by comparing against so-called ‘gold standard’ parameters, which are based on studies performed in single laboratories and not substantiated by the overall published literature. Our analysis also highlights the need for minimal reporting guidelines in the field of experimental atherosclerosis, as 29 of the 204 research papers reported only one of the basic parameters that we evaluated.

**Conflict of interest:** none declared.

### Funding

This study was funded by the British Heart Foundation (PG/16/44/32146) and by a Marie-Sklodowska Curie ITN (721532) award from the European Union.
